# Social anxiety disorder and antibodies against glial cells in the cerebrospinal fluid

**DOI:** 10.1016/j.bbih.2025.101064

**Published:** 2025-07-19

**Authors:** Judith Weiser, Alexander Rau, Katharina von Zedtwitz, Bernd Feige, Kathrin Nickel, Simon J. Maier, Raphael J. Dressle, Nils Venhoff, Ludger Tebartz van Elst, Miriam A. Schiele, Katharina Domschke, Harald Prüss, Dominique Endres

**Affiliations:** a Department of Psychiatry and Psychotherapy, Medical Center - University of Freiburg, Faculty of Medicine, University of Freiburg, Freiburg, Germany; b Department of Neuroradiology, Medical Center - University of Freiburg, Faculty of Medicine, University of Freiburg, Freiburg, Germany; c Department of Rheumatology and Clinical Immunology, Medical Center - University of Freiburg, Faculty of Medicine, University of Freiburg, Freiburg, Germany; d German Center for Mental Health (DZPG), Partner Site Berlin/Potsdam, Berlin, Germany; e Department of Neurology and Experimental Neurology, Charité - Universitätsmedizin Berlin, Berlin, Germany; f German Center for Neurodegenerative Diseases (DZNE) Berlin, Berlin, Germany

**Keywords:** Social phobia, Anxiety, Inflammation, Autoimmune, Recoverin

## Abstract

**Introduction:**

Autoimmune social anxiety disorders have not yet been described in the literature.

**Methods:**

Therefore, this case of a patient with possible autoimmune-mediated social anxiety disorder is presented. Due to treatment resistance and high serum streptococcal antibody levels, a comprehensive diagnostic work-up was performed.

**Results:**

The 19-year-old female patient presented with predominant social anxiety disorder and secondary depression. Testing for all known characterized neuronal and glial IgG antibodies identified slightly positive (“+”) recoverin IgG antibodies only in the serum (using an immunoassay). Cerebrospinal fluid (CSF) analysis using a tissue-based assay on unfixed mouse brain slices revealed moderate immunoglobulin G (IgG) anti-nuclear binding and a specific strong IgG binding against cell nuclei of the Bergmann glia in the cerebellum. No clear pathology was noted in conventional magnetic resonance imaging (MRI), voxel-based morphometry, and cerebral blood flow. Diffusion microstructure imaging (DMI) revealed a substantial reduction of the intraneurite volume fraction in the cerebellar gray and white matter. This was accompanied by a compensatory increase in free fluid and the extra-neurite volume fraction. Alterations in the striatum were also observed with DMI. Electroencephalography (EEG) showed intermittent generalized slowing with underlying left frontal, right temporal, and left temporo-occipital components (detected via independent component analysis of the EEG). The [^18^F]fluorodeoxyglucose positron emission tomography of the whole body detected a slight polyserositis and no malignant tumor.

**Discussion:**

This is the first description of a case with evidence of a possible antibody-mediated cerebellar dysfunction associated with social anxiety disorder. The testing for all characterized neuronal/glial antibodies showed only weakly positive recoverin antibodies in the serum, which, however, were not detectable in the CSF. Therefore, novel CSF antibodies directed against cell nuclei of the Bergmann glia in the cerebellum could be assumed. DMI alterations in the cerebellum were in principle compatible with neuroinflammation with edematization and cellular activation. In addition, the further DMI alterations in the striatum and the fronto-temporal generators of EEG slowing suggest an involvement of the “anxiety network”. Further immunopsychiatric research in anxiety disorders might contribute to identifying an autoimmune subtype and potentially immunomodulatory treatment options.

## 1 Introduction

Autoimmune-mediated inflammatory processes in anxiety disorders have been increasingly investigated recently (Michopoulos et al., 2017; Goldsmith et al., 2023; Sah and Singewald, 2025). Neurological conditions such as stiff-person syndrome, which is associated with glutamic acid decarboxylase 65 antibodies, are often accompanied by anxiety symptoms (Graus et al., 2020; Bose and Jacob, 2025). In panic disorder, altered cytokine profiles have been described (Quagliato and Nardi, 2022), which might be targeted by psychotherapy (Ziegler et al., 2019). An autoimmune glial hypothesis has been widely discussed in the context of depression (Xie et al., 2022; Lu et al., 2025). In clinical neurology, paraneoplastic cerebellar degeneration and autoimmune encephalitis are well established disorders (Graus et al., 2016; Lewerenz et al., 2016). A number of preclinical studies have suggested a particular role of microglia also in governing anxiety-like behavior (McKim et al., 2018; Biltz et al., 2024; Huang et al., 2024), and the cerebellum has been implicated in integrating exteroceptive and proprioceptive anxiety-related information (Hilber, 2022). A systematic literature search on PubMed with the following search strategy, “(anxiety AND cerebellum AND antibody),” performed on March 2nd, 2025, yielded 17 results, but no published cases with autoimmune-mediated social anxiety disorder. However, one case with Tr-antibody positive cerebellar ataxia and additional mild anxiety symptoms was identified (Shen et al., 2021)

The present article describes the first case of a possible autoimmune-mediated social anxiety disorder with glial antibodies in cerebrospinal fluid (CSF) detected using a tissue-based screening assay and cerebellar involvement.

## 2 Methods

The patient gave written informed consent for the publication of this case report. The diagnostic work-up followed a protocol developed for the detection of secondary obsessive–compulsive syndromes and incorporating a comprehensive laboratory work-up including testing for streptococcal antibodies and rheumatological screening, magnetic resonance imaging (MRI), and electroencephalography (EEG) (Runge et al., 2023). In addition, a routine analysis of the CSF and testing for several immunoglobulin G (IgG) central nervous system (CNS) autoantibodies were carried out. Testing for CNS antibodies in the local CSF laboratory (https://www.uniklinik-freiburg.de/neurologie/klinik/diagnostische-einrichtungen/liquor-labor.html) included testing for neuronal antibodies against the cell surface antigens AMPA-1/2-receptor(R)/CASPR2/DPPX/GABA-B-R/LGI1/NMDA-R using a fixed cell-based assay (Mosaic 6, Euroimmun®, Lübeck, Germany) in serum and CSF, against the intracellular antigens amphiphysin/CV2(CRMP5)/GAD65/Hu/Ma1/Ma2/Ri/SOX1/TR(DNER)/Yo/Zic4 using an immunoblot in serum (Ravo PNS 11 Line Assay®, Freiburg, Germany), and against the glial antigens MOG/AQP4 in serum using a fixed cell-based assay (Euroimmun®, Lübeck, Germany) (Endres et al., 2022). Screening for novel CNS antibodies was performed using a tissue-based assay (Kreye et al., 2020; Franke et al., 2021; Endres et al., 2022). For this, unfixed mouse brain tissue sections were after preparation incubated overnight with diluted serum (1:200 dilution) and undiluted CSF (1:1 dilution). After further washing steps, a semi-quantitative fluorescence rating was carried out. The methodology of this tissue-based assay was previously published in detail (Kreye et al., 2020; Franke et al., 2021; Endres et al., 2022).

In addition, a broad range of already characterized, commercially testable CNS autoantibodies were examined in an external laboratory (Stöcker laboratory, Groβ-Gronau, Germany) using serum and CSF samples. These analyses were performed using immunoblots and immunofluorescence tests (IFT) on fixed biochip mosaics with brain tissue and recombinant cells (for further details see: https://www.labor-stoecker.com/wp-content/uploads/pdf-downloads/lv_autoimmun_de.pdf; Euroimmun®, Lübeck, Germany). All antibodies tested are summarized in Table 1. The EEG was also analyzed automatically using an independent component analysis (Endres et al., 2017). Apart from conventional structural MRI, advanced MRI analyses included automated morphometry (VEOmorph®; https://www.veobrain.com/?page=veomorph), diffusion tensor imaging (DTI) with diffusion microstructure imaging (DMI), and arterial spin labeling (ASL) (Rau et al., 2022; Hosp et al., 2024). The DTI, DMI, and ASL MRI data of the patient were compared with a sex (only female healthy controls) matched control group of 15 healthy subjects with comparable age (24.6 ± 2.35 years) (Rau et al., 2022; Hosp et al., 2024). Z-scores were calculated and findings with Z-scores <3 and >3 were reported. A Z-score threshold of 3 was chosen based on its statistical properties in the context of a normal distribution, where values beyond ±3 standard deviations represent the extreme 0.3 % of the data. This conservative cutoff helps to highlight only the most pronounced deviations from the norm while minimizing the inclusion of features that may arise from noise or natural variance. Given the large number of extracted features and the risk of false positives, we selected this threshold to ensure high specificity in identifying relevant imaging abnormalities. Furthermore, [^18^F]fluorodeoxyglucose positron emission tomography (FDG-PET) of the brain and whole body was performed. Ophthalmological examinations included optical coherence tomography (OCT) and electroretinogram (ERG). Psychometric testing comprised the Social Interaction Anxiety Scale (SIAS) and the Social Phobia Scale (SPS) (Mattick and Clarke, 1998).

**Table 1:** All diagnostic findings at baseline. *Pathological findings are highlighted in bold italics****.***

Parameters	Results in the presented case
Blood tests	
Systemic serum autoantibodies and immunological markers	
Anti-thyroid antibodies (against TPO, TG and TSH-receptor)	Negative
ANAs (*on HEp-2 cells*), ANCAs (*on EthOH-/formalin-fixed neutrophils*), APAs, dsDNA antibodies	Negative
CCP, rheumatoid factor	Normal
Gliadin and transglutaminase antibodies	Negative
Complement factors (C3, C4, CH50)	Normal
IgG, IgM and IgA levels	Normal
CRP	Normal
Sarcoidosis parameters (IL-2 R, neopterin, ACE)	Normal
Serologies	
Serology for Lyme disease, lues, CMV, EBV, HBV, HCV, HIV, toxoplasmosis, tuberculosis	***EBV-EBNA-1 IgG positive as an indication of excluded acute infection in case of seropositivity.*** Everything else negative.
Streptococcal antibodies	
Streptococcus DNAse B antibodies	***747 U/ml*** (ref.: <200 U/ml)
Streptolysin O antibody	***322 U/ml*** (ref.: <300 U/ml)
Serum neuronal autoantibodies	
Paraneoplastic IgG antibodies against intracellular antigens (amphiphysin, CV2(CRMP5), GAD65, Hu, Ma1, Ma2, Ri, SOX1, TR(DNER), Yo, Zic4)	Negative
Well-characterized neuronal IgG cell surface antibodies (AMPA-1/2-R, CASPR2, DPPX, GABA-B-R, LGI1, NMDA-R)	Negative
Anti-MOG/AQP4/-IgG antibodies	Negative
Tissue based assay on unfixed murine brain tissue (Prof. Prüss, Charité Berlin, Germany)	***Moderate global binding to anti-nuclear structures*.**
Extended neuronal and glial autoantibody testing in serum (Stöcker laboratory, Groβ-Gronau, Germany):	
*Neuronal IgG autoantibodies using immunoblots (Euroimmun®, Lübeck, Germany):*	
Neuronal IgG autoantibodies in the immunoblot for amphiphysin, CV2, PNMA2, Ri, Yo, Hu, recoverin, SOX1, titin, Zic4, GAD65, and DNER (PNS12/145-59, Paraneoplastic neurological syndromes Euroline® IgG)	***Recoverin IgG antibodies positive (“+”)***
Neuronal IgG autoantibodies in the immunoblot for Yo, CDR2L, DNER, PRKCG, ARHGAP26, Homer-3, RGS8, RYR2, and AP3B2 (PCP/06–12, Purkinje Euroline® IgG)	Negative
*Neuronal IgG autoantibodies in IFT (Euroimmun®, Lübeck, Germany):*	
GFAP IgG autoantibodies in IFT	Negative
Standard immunofluorescence tests for the following autoantibodies: Hu, Ri, ANNA-3, Tr/DNER, Ma (Ma1, Ma2/Ta), GAD65, amphiphysin, aquaporin-4, MOG, glutamate receptors (NMDA type), glutamate receptors (AMPA type), GABA-B receptors, LGI1, CASPR2, IgLON5, and DPPX	Negative
Autoantibodies against myelin	Negative
*Further (non-accredited) tests on IFT (Euroimmun®, Lübeck, Germany):*	Negative
Autoantibodies against CARPVIII, glycine receptors, mGluR1, mGluR5, GABA-A receptors, Rho GTPase activating protein 26, recoverin, GluRD2, flotillin-1/2, and Zic4	Negative
Autoantibodies against ITPR1, Homer 3, neurochondrin, and neurexin-3-alpha	
Autoantibodies against ERC1, Sez6I2, AP3B2, contactin1, neurofascin 155/186, AT1A3, KCNA2, and dopamine receptor 2	
**Cerebrospinal fluid**	
White blood cell count	2/μL (ref.: <5/μL)
Protein concentration	414 mg/L (ref.: <450 mg/L)
Albumin quotient	4.8 (ref.: <5)
IgG-index	0.4 (ref.: <0.7)
Oligoclonal bands in serum/CSF	Negative/negative
Local IgG/IgA/IgM synthesis	No
Well-characterized neuronal IgG cell surface antibodies (AMPA-1/2-R, CASPR2, DPPX, GABA-B-R, LGI1, NMDA-R)	Negative
Tissue based assay on unfixed murine brain tissue (Prof. Prüss, Charité Berlin, Germany)	***Moderate global binding to anti-nuclear structures. Strong IgG binding against cell nuclei of the Bergmann glia (astrocytes) in the cerebellum.***
Extended neuronal and glial autoantibody testing in cerebrospinal fluid (Stöcker laboratory, Groβ-Gronau, Germany):	
*Neuronal IgG autoantibodies using immunoblots (Euroimmun®, Lübeck, Germany):*	
Neuronal IgG autoantibodies in the immunoblot for amphiphysin, CV2, PNMA2, Ri, Yo, Hu, recoverin, SOX1, titin, Zic4, GAD65, and DNER (PNS12/119-21, Paraneoplastic neurological syndromes Euroline® IgG)	Negative
Neuronal IgG autoantibodies in the immunoblot for Yo, CDR2L, DNER, PRKCG, ARHGAP26, Homer-3, RGS8, RYR2, and AP3B2 (PCP/07–41, Purkinje Euroline® IgG)	Negative
*Neuronal IgG autoantibodies in IFT (Euroimmun®, Lübeck, Germany):*	
GFAP IgG autoantibodies in IFT	Negative
Standard immunofluorescence tests for the following autoantibodies: Aquaporin-4, glutamate receptors (NMDA type), GABA-B receptors, and CASPR2	Negative
*Further (non-accredited) tests on IFT (Euroimmun®, Lübeck, Germany):*	
Autoantibodies against Hu, Ri, ANNA-3, Yo, Tr/DNER, myelin, Ma (Ma1, Ma2/Ta), GAD65, amphiphysin, glutamate receptors (AMPA type), LGI1, Zic4, DPPX, CARPVIII, glycine receptors, mGluR1, mGluR5, GABA-A receptors, Rho GTPase activating protein 26, ITPR1, Homer 3, MOG, recoverin, neurochondrin, GluRD2, flotillin-1/2, and IgLON5	Negative
Autoantibodies against neurexin-3-alpha, ERC1, Sez6I2, AP3B2, contactin1, neurofascin 155/186, AT1A3, KCNA2, and dopamine receptor 2	Negative
**MRI of the neurocranium**	
Conventional MRI	Normal
**EEG**	
Visual analysis	***Intermittent rhythmic generalized slowing.***
Independent component analysis	***IRDA/IRTA rates of 6 per minute, increased after HV. There is primarily a left frontal component, further right temporal and left temporo-occipital. In some of the events a generalization over many components can be observed; occasionally there is a spike-wave on the left frontal side. Spike-wave-like patterns are also observed on the left frontal side. After HV, the EEG signal is clearly more disturbed.***
**FDG-PET**	
Brain	Normal.
Whole body	No evidence of malignant hypermetabolic lesions; however, ***there were slight pleural effusions, a discrete pericardial effusion, and a discrete ascites in the small pelvis.***
**Ophthalmological tests**	
Optical coherence tomography	Normal
Electroretinography	***Slight dysfunction of the cones***, no clear indication of a retinopathy.
**Further investigations**	
Transthoracic echocardiography	***Minimal pericardial space in front of the left ventricle***, otherwise unremarkable.
Sonography	***Minimal non-puncturable pleural effusions on both sides***, no ascites
Protein and albumin levels in serum	Normal

Abbreviations: ACE, angiotensin-converting enzyme; AMPA, α-amino-3-hydroxy-5-methyl-4-isoxazolepropionic acid; ANAs, antinuclear antibodies; ANCAs, anti-neutrophil cytoplasmic antibodies; ANNA-3, Antineuronal Nuclear Antibody Type 3; MOG, Myelin oligodendrocyte glycoprotein; APAs, Antiphospholipid antibodies; AP3B2, Adaptor-related protein complex 3 beta 2 subunit; AQP4, Aquaporin-4; AP3B2, Adaptor Related Protein Complex 3 Subunit Beta 2; ARHGAP26, Rho GTPase Activating Protein 26; ATP1A3, ATPase Na+/K + Transporting Subunit Alpha 3; CCP, Cyclic citrullinated peptide; CASPR2, Contactin-associated protein-like 2**;** CMV, Cytomegalovirus; CRP, C-reactive protein; CSF, Cerebrospinal fluid; DNaseB, Deoxyribonuclease B; DNER, Delta/Notch Like EGF Repeat Containing; DPPX, Dipeptidyl-peptidase–like protein; dsDNA, Double stranded DNA; EBV, Epstein-Barr virus; EEG, Electroencephalography; FDG-PET, [^18^F]fluorodeoxyglucose positron emission tomography; GAD65, Glutamic acid decarboxylase 65-kDa isoform; GABA, Gamma-aminobutyric acid; GFAP, Glial fibrillary acidic protein; GRID2, Glutamate Ionotropic Receptor Delta Type Subunit 2; HBV, Hepatitis B virus; HCV, Hepatitis C virus; HIV, human immunodeficiency virus; IFT, immunofluorescence tests; ITPR1, Inositol trisphosphate receptor 1; IFT, Immunofluorescence testing**;** IgA/G/M, Immunoglobulin A/G/M; IL-2 R, Interleukin-2 receptor; IRDA, Intermittent rhythmic delta activity; LGl1, Leucine-rich glioma-inactivated 1; IgLON5, IgLON family member 5 condition**;** MGLur5, Metabotropic glutamate receptor 5; MOG, myelin oligodendrocyte glycoprotein; MRI, magnetic resonance imaging; NMDA, N-methyl-D-aspartate; PNMA2, paraneoplastic Ma antigen2; PRKCG, Protein kinase C gamma; RGS8, Regulator of G-protein signaling 8**;** RYR2, Ryanodine receptor 2; SEZ6L2, Seizure Related 6 Homolog Like 2; TG, thyroglobulin; TPO, thyroid peroxidase; TSH, thyroid-stimulating hormone; ZIC4, Zinc-finger protein 4.

## 3 Results

The 19-year-old female patient presented with a predominant severe social anxiety disorder, which had worsened over the past two years and was characterized by a strong feeling of discomfort in groups of people and a fear of embarrassing herself. She was unable to approach people she did not know. Social situations such as shopping were completely avoided. Psychometric testing (outside of the depressive episode, at the end of the inpatient treatment) revealed still increased scores on the SIAS (43 of 80 points; cut off ≥30 points) and SPS (24 of 80 points; cut off ≥24 points). Due to a low capacity for introspection (according to our clinical assessment), there was a difference in the clinical external judgment and the questionnaire scores completed by the patient, with a more severe manifestation in the clinical assessment. During inpatient treatment she developed a secondary severe depressive episode. An underlying autism spectrum disorder was evaluated, but the clinical criteria were not fulfilled; in particular, there were no evident social communication deficits beyond shyness, nor were there any clear routines, rituals, or special interests. The patient exhibited only isolated autism spectrum symptoms (i.e., sometimes “swinging” with the leg and sensory overload for loud noises). No somatic diseases were pre-diagnosed, and the neurological examination was unremarkable with no signs of cerebellar dysfunction. The family history was inconspicuous for mental illnesses. Antidepressant treatment with sertraline failed to sufficiently improve social anxiety disorder. Exposure-based cognitive behavioral therapy was only partially possible due to the severity of the symptoms and worsening of the depression during guideline-based therapy. Augmentative treatment with low-dose olanzapine and lithium was carried out due to severe depressive exacerbation, leading to an improvement in depressive symptoms. Recent outpatient treatment with escitalopram and mirtazapine yielded no relevant improvement in social anxiety or depressive symptoms.

**Fig. 1:**
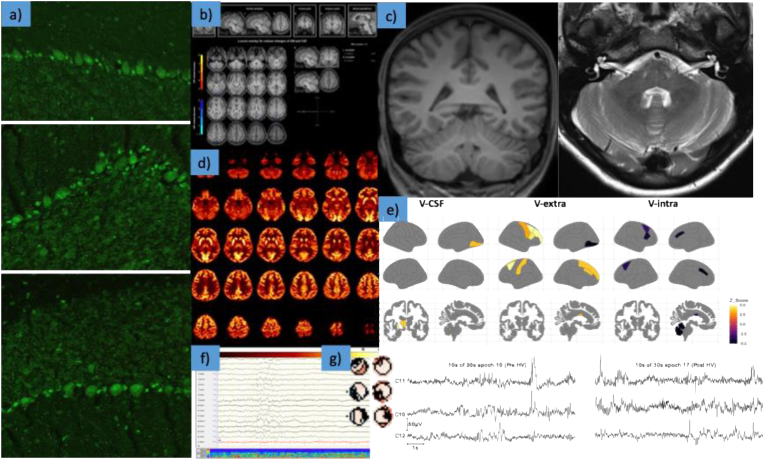
Graphical illustrations of diagnostic findings at baseline. a) Immunoglobulin G (IgG) antibodies against cell nuclei of the Bergmann glia in the cerebellum detected in cerebrospinal fluid (CSF) samples of the patient. The nuclei of the Bergmann glial cells are clearly visible between the larger Purkinje cells. b) Automated morphometry (using VEOmorph software; https://www.veobrain.com/?page=veomorph) revealed slight occipital but no cerebellar volume loss. c) Conventional magnetic resonance imaging (MRI) showed no clear cerebellar volume loss (at most one could assume accentuated cerebellar foliae). There were also no further macroscopic abnormalities. d) [^18^F]fluorodeoxyglucose positron emission tomography of the brain revealed normal findings. e) Diffusion microstructural imaging (DMI) changes in MRI identified a substantial reduction of the intraaxonal and –dendrite volume fraction (V-intra) in the cerebellar gray and white matter. This was accompanied by a compensatory increase in free fluid (V-CSF) and the cellular and extracellular volume fraction (V-extra). Further DMI changes were detected in the left pallidum, left thalamus, left caudate, and central corpus callosum (see also Table 2). f) Intermittent generalized slowing in electroencephalography (EEG). g) Independent component analyses of the EEG detected frontal, right temporal, and left temporo-occipital components; pre- and post-hyperventilation (HV) state is presented.

The diagnostic work-up revealed strongly increased streptococcal DNase B antibodies in the serum, as well as anti-nuclear antibodies of moderate intensity in the tissue-based assay in serum and CSF. In addition, the CSF samples showed strong IgG antibody binding against cell nuclei of the Bergmann glia in the cerebellum. Testing for all characterized brain antibodies revealed only positive recoverin IgG antibodies in serum with weak intensity in the immunoassay (+; reference: negative; range: 0, (+), +, ++, +++). CSF testing for recoverin IgG antibodies using an immunoassay (as well as recoverin antibody testing in both serum and CSF using fixed cell-based assays) remained negative. The EEG identified intermittent generalized slowing. ICA of the EEG detected components localized to the left frontal, right temporal, and left temporo-occipital area with occasional spike-wave-like patterns observed in the left frontal area. No clear macrostructural pathology was noted in conventional MRI of the brain. DMI revealed a marked reduction of the intraneurite volume fraction in both cerebellar gray and white matter. This was accompanied by a compensatory increase in free fluid and the extraneurite volume fraction. In addition, DMI changes were detected in the left pallidum, left thalamus, left caudate, and central corpus callosum. DTI showed alterations in fractional anisotropy of the mid-anterior corpus callosum, possibly affecting network connectivity. Additional changes in the fractional anisotropy of the left lateral ventricle were interpreted as a partial volume effect. ASL detected no changes in cerebral blood flow, and automated morphometry showed only slight bilateral occipital gray matter volume decrease without evidence of cerebellar atrophy (Table 2). FDG-PET identified normal cerebral glucose metabolism. Whole-body FDG-PET showed no evidence of malignant hypermetabolic lesions, although mild polyserositis was observed. The rheumatological, pneumological and gynecological examinations revealed no evidence of rheumatological disease or a malignant tumor. Ophthalmological examinations, including OCT and ERG, showed a slight dysfunction of the cones, yet, there was no autoimmune retinopathy. An immunotherapeutic treatment trial with methylprednisolone was recommended; however, the patient did not consent the treatment at this time. The diagnostic findings are summarized in Table 1, Table 2 and Fig. 1.

**Table 2:** Advanced magnetic resonance imaging (MRI) included diffusion microstructure imaging (DMI), diffusion tensor imaging (DTI), arterial spin labeling (ASL), and automated morphometry. All cerebellar changes are highlighted in red. The MRI data of the case study patient were compared with a sex matched control group of 15 female healthy individuals with comparable age (24.6 ± 2.35 years) after parcellation according to established brain atlases (Whole Brain Segmentation: Automated Labeling of Neuroanatomical Structures in the Human Brain; Fischl et al., 2002) and using an automated labeling system for subdividing the human cerebral cortex on MRI scans into gyral based regions of interest (Desikan et al., 2006). DMI parameters included 1) the free water/cerebrospinal fluid (CSF) fraction (V-CSF), in which the molecules move randomly at the distance of their diffusion length; 2. the volume fraction (V-intra) within neuronal processes, i.e. axons and dendrites, with almost one-dimensional molecular diffusion at narrow membrane boundaries; and 3. the volume fraction of the cellular compartment and the extracellular matrix (V-extra) outside the axons or dendrites, which is characterized by an average restriction of molecular diffusion. Diffusion tensor imaging (DTI) parameters included fractional anisotropy (FA), axial diffusivity (AD), and mean diffusivity (MD). Arterial spin labeling (ASL) analyzed cerebral blood flow. Automated morphometry analyzed gray matter (GM) volume decrease and CSF volume increase using a commercially available software (VEOmorph®; https://www.veobrain.com/?page=veomorph).

Parameter	Region	Z-Score (Ref. >3 and < 3)
**Diffusion Microstructure Imaging (DMI)**		
V-CSF	Left cerebellar white matter	4.3
V-CSF	Left pallidum	4.8
V-CSF	Left thalamus	3.7
V-CSF	Right cerebellar white matter	3.0
V-extra	Central corpus callosum	3.2
V-intra	Central corpus callosum	3.2
V-intra	Left caudate	−3.5
V-intra	Left cerebellar cortex	−3.5
V-intra	Left cerebellar white matter	−4.1
V-intra	Right cerebellar cortex	−4.7
**Diffusion Tensor Imaging (DTI)**		
DTI-FA	Corpus callosum mid-anterior	−3.1
DTI-FA	Left lateral ventricle	3.7
**Arterial Spin Labeling (ASL)**		
No pathologies in cerebral blood flow		
**Automated Morphometry (VEOmorph®)**		
GM-volume decrease	Occipital (cuneus) left	5.0
GM-volume decrease	Occipital (cuneus) right	4.6

## 4 Discussion

A patient with antibodies against cell nuclei of the Bergmann glia in the CSF, cerebellar and striatal microstructural MRI alterations, and generalized EEG slowing with fronto-temporal components along with social anxiety disorder was described.

***From a clinical perspective***, especially the generalized EEG slowing and the mild polyserositis were suggestive of a secondary cause. Extensive neuronal/glial antibody testing provided evidence of a possible antibody-associated process. It can be speculated whether a streptococcal infection, with elevated streptococcal antibodies, triggered B cells to produce glial antibodies.

***From a pathophysiological perspective***, this case indicates that antibodies against astrocytes can be associated not only with classical astrocytopathy (Flanagan et al., 2017) and perhaps depression (Endres et al., 2023a), but possibly also with social anxiety disorder; whereby antibodies against cell nuclei of the Bergmann glia were found here and no evidence of the more common astrocytic Bergmann glia fiber staining, as described in a case of autoimmune depression (Endres et al., 2023a, Endres et al., 2023b) or in autoimmune astrocytopathies (Flanagan et al., 2017). The intracellular antigen localization might explain the “milder” disease course. Compared to the pathophysiological processes of neuronal antibodies against cell surface antigens (e.g., against the NMDA-R), antibodies against intracellular antigens encounter increased difficulty in reaching their antigen target, as they first have to enter the intracellular compartment as here with the cell nuclei of the Bergmann glia as the target (Endres et al., 2023b; Maier et al., 2024). The intrathecally dominant antibody reactivity was a sign of intrathecal immune activation. Recoverin antibodies were weakly positive in serum using an immunoassay. Recoverin is a calcium-binding photoreceptor protein of the retina that is involved in the transmission of light (Kaiser, 1999; Bataller and Dalmau, 2004). Antibodies against recoverin are mostly found in paraneoplastic, autoimmune retinopathy (Chen et al., 2021) and associated malignancies are mainly small cell lung carcinoma and occasionally gynecological tumors (Kaiser, 1999; Bataller and Dalmau, 2004); in this patient retinopathy and malignancies were ruled out. The recoverin antibodies were negative in the fixed cell-based assay in both serum and CSF, and the immunoassay was also negative using CSF samples, so it was not expected that the antibodies against cell nuclei of the Bergmann glia in CSF were directed against recoverin. Therefore, a novel glial antibody could be assumed. Advanced MRI supported the hypothesis of functional antibody effects due to microstructural changes in the cerebellum which would in principle be compatible with antibody-associated neuroinflammation with edematization and cellular activation (Spanò et al., 2018). In addition, supporting our findings, preclinical studies suggested a relevant role of microglia in the control of anxiety-like behavior (McKim et al., 2018; Biltz et al., 2024; Huang et al., 2024), and a recent study indicated that acute stress-induced anxiety-like behaviors may result from interactions between microglial cells and altered GABA concentrations (Chen et al., 2024). Suspected antibody-mediated cerebellar dysfunction would be consistent with a “cerebellar cognitive affective syndrome” (Schmahmann and Sherman, 1998). Ito has proposed that the cerebellum controls mental activities by internal models (Ito, 2006, 2008) and disturbances in these internal cerebellar models may also affect the “anxiety network” (Abi-Dargham et al., 2023; Hilber, 2022). This network includes altered activity in the orbitofrontal, dorsolateral, dorsomedial, and ventrolateral prefrontal cortex as well as increased activity in limbic areas such as the amygdala, the insula, the anterior cingulate cortex, the striatum, and the bed nucleus of the stria terminalis (Abi-Dargham et al., 2023). In the presented patient, further structural alterations in the striatum (more precisely in the left caudate and pallidum) including parts of the “anxiety network” were detected using DMI. Furthermore, the fronto-temporal generators of EEG slowing suggest an involvement of the “anxiety network” on the electrophysiological level. It is therefore conceivable that the antibodies against cerebellar structures have influenced neuronal circuits at the network level that are associated with social anxiety symptoms.

***A limitation*** of the study is that this was only one complex psychiatric syndrome with secondary depression and no well-established signs of neuroinflammation in conventional MRI, CSF or FDG-PET were detected, raising the possibility that social anxiety disorder could have developed completely independent of the glial antibodies. However, “less severe” psychiatric syndromes such as anxiety disorders with an underlying autoimmune process are also more likely to be associated with subtle inflammatory activity, as they are less fulminant than autoimmune encephalitis. Recently, in two case studies, an association of recoverin antibodies and cerebellitis were reported (Herzog et al., 2020; Minetti et al., 2023), so that it cannot be fully ruled out that the detected CSF antibodies might be directed against recoverin.

**In summary**, this case provides the first evidence that antibody-mediated cerebellar dysfunction might be associated with social anxiety disorder. In the next step, the underlying antigens and the functionality of similar antibodies should be investigated to prove a possible causality. Antigen targets could be identified using methods such as immunoprecipitation-mass spectrometry. Their functionality could be studied by injecting the purified antibodies intrathecally into mouse brains via pumps, followed by behavioral experiments testing whether the symptoms observed in human patients are also triggered by the respective antibodies in the mouse model (Prüss, 2021). Future immunopsychiatric research could potentially result in novel therapeutic options for anxiety disorders involving immunomodulatory treatments such as microglia-directed treatments, pro-inflammatory cytokine inhibitors or COX-inhibitors (Sah and Singewald, 2025).

## CRediT authorship contribution statement

**Judith Weiser:** Writing – original draft, Visualization, Methodology, Investigation, Formal analysis, Data curation, Conceptualization. **Alexander Rau:** Writing – review & editing, Methodology, Formal analysis, Data curation. **Katharina von Zedtwitz:** Writing – review & editing, Investigation, Data curation. **Bernd Feige:** Writing – review & editing, Methodology, Formal analysis. **Kathrin Nickel:** Writing – review & editing, Methodology, Data curation. **Simon J. Maier:** Writing – review & editing, Methodology, Formal analysis. **Raphael J. Dressle:** Writing – review & editing, Visualization, Investigation, Data curation. **Nils Venhoff:** Writing – review & editing, Investigation. **Ludger Tebartz van Elst:** Writing – review & editing, Supervision. **Miriam A. Schiele:** Writing – review & editing, Methodology, Formal analysis. **Katharina Domschke:** Writing – review & editing, Supervision, Resources. **Harald Prüss:** Writing – review & editing, Visualization, Supervision, Methodology, Investigation, Formal analysis, Data curation. **Dominique Endres:** Writing – original draft, Visualization, Supervision, Investigation, Formal analysis, Data curation, Conceptualization.

## Ethics/consent for publication

The patient has given her signed written informed consent for this case report to be published.

## Availability of data and material

All necessary data can be found in the paper.

## Funding

DE, BF and LTvE were funded by the German Research Foundation (Project-Nr.: 419859038). DE was additionally supported by the German Ministry of Education and Research (01GM2208, CONNECT-GENERATE).

## Declaration of competing interest

KD: Member of the Neurotorium Editorial Board, The Lundbeck Foundation. She received speaker's honoraria by Janssen-Cilag GmbH. LTvE: Advisory boards, lectures, or travel grants within the last three years: Roche, Eli Lilly, Janssen-Cilag, Novartis, Shire, UCB, GSK, Servier, Janssen, and Cyberonics. All other authors declare no potential conflicts of interest.

## Data Availability

This is a case study. All necessary data can be found in the manuscript.
